# Evaluation of a miniaturized NIR spectrometer for cultivar identification: The case of barley, chickpea and sorghum in Ethiopia

**DOI:** 10.1371/journal.pone.0193620

**Published:** 2018-03-21

**Authors:** Frédéric Kosmowski, Tigist Worku

**Affiliations:** CGIAR Standing Panel on Impact Assessment, ILRI, Addis Ababa, Ethiopia; The University of Sydney, AUSTRALIA

## Abstract

Crop cultivar identification is fundamental for agricultural research, industry and policies. This paper investigates the feasibility of using visible/near infrared hyperspectral data collected with a miniaturized NIR spectrometer to identify cultivars of barley, chickpea and sorghum in the context of Ethiopia. A total of 2650 grains of barley, chickpea and sorghum cultivars were scanned using the SCIO, a recently released miniaturized NIR spectrometer. The effects of data preprocessing techniques and choosing a machine learning algorithm on distinguishing cultivars are further evaluated. Predictive multiclass models of 24 barley cultivars, 19 chickpea cultivars and 10 sorghum cultivars delivered an accuracy of 89%, 96% and 87% on hold-out sample. The Support Vector Machine (SVM) and Partial least squares discriminant analysis (PLS-DA) algorithms consistently outperformed other algorithms. Several cultivars, believed to be widely adopted in Ethiopia, were identified with perfect accuracy. These results advance the discussion on cultivar identification survey methods by demonstrating that miniaturized NIR spectrometers represent a low-cost, rapid and viable tool. We further discuss the potential utility of the method for adoption surveys, field-scale agronomic studies, socio-economic impact assessments and value chain quality control. Finally, we provide a free tool for R to easily carry out crop cultivar identification and measure uncertainty based on spectral data.

## Introduction

Crop cultivar identification is an important issue for both agricultural research and policies. Progress toward agricultural intensification and value chains development policies in sub-Saharan Africa must rely on valid measurements to be successful [1]. Furthermore, a large body of scholarship relies on empirical measures of cultivars. Examples include assessing the on-farm impact of newly developed cultivars [2, 3], estimating adoption rates of cultivars at the national level [4, 5] or developing recommendation areas for future crop dissemination efforts based on agro-ecological variables [6]. In addition, cultivar identification is also of technical and economic importance for the agricultural industry. At different stages of the value chain, assessment of cultivar uniformity is often crucial for the production of high quality seeds or to match consumer preferences.

There is no doubt that estimating modern cultivar adoption is a challenging task. Currently, household surveys relying on farmers’ elicitation represent the most common source of data [5, 7]. Various other methodologies, such as sales inquiries or expert opinion estimates have also been employed [4]. However, assessing the extent of measurement errors was impossible in the absence of an objective benchmark. Since 2010, the technology of DNA fingerprinting has become increasingly affordable, providing an opportunity to conduct field survey validation exercises in a variety of contexts. Recent findings on various crops have demonstrated that no other method can match the accuracy of DNA fingerprinting [8–11]. However, DNA fingerprinting remains costly and time-consuming to implement and can pose some logistical challenges. Thus, there is a strong interest in exploring alternative methods of data collection that could be used in combination.

Near-infrared (NIR) spectrometry -–a technique that collects the reflected light of a sampled material in the near-infrared region of the electromagnetic spectrum–is an alternative method that can deliver information on the biological composition and surface characteristics of grains. Each type of molecule vibrates in its own unique way; and molecular vibrations interact with light to create a unique spectral signature. The main sources of spectral variance include grain size (large kernels reflecting more variations), grain shape and curvature [12] as well as grain position and orientation during scanning. Spectroscopy has been previously applied for varietal identification of rice [13–15], maize [16–17], wheat [18–19] and soybean [20]. Several other studies have used the technique to assess the content and quality of a wide diversity of agricultural products [21]. Analyses are generally performed by specialized laboratories and rely on different devices that generate spectral data with different wavelength ranges. As mentioned by [22], available knowledge in the recent scientific literature has tended to rely on partial least squares discriminant analysis (PLS-DA) under laboratory conditions, using a small number of varieties only. It is currently unclear whether NIR spectrometry could represent a valuable and scalable tool for cultivar identification in sub-Saharan Africa.

Recently, miniaturized, lower cost NIR spectrometers have been commercialized and could represent an alternative to laboratory-based spectral measurements. [23] demonstrate the practical applicability of such devices for detecting falsified artemisinin-based malaria medicines. While these spectrometers have a lower resolution than laboratory-based devices, the possibility to generate more data at a reduced cost, coupled with the use of machine learning methods, could potentially offset this disadvantage. Applied in numerous fields of research, methods such as Random Forest or Support Vector Machines have demonstrated their superior accuracy for classification models [15, 22].

To our knowledge, no studies have assessed the feasibility of this approach in the field of agricultural research. Here, we propose to evaluate a miniaturized spectrometer device for discriminating cultivars of barley, chickpea and sorghum in Ethiopia. These three crops were chosen for their important contribution to the Ethiopian agricultural system as well as their different morphological attributes in terms of shape, size, texture and colour. Barley grains have an elongated shape and are divided longitudinally in half by a crease extending over the whole length of the grain. Grains are pale yellow, with very low levels of distinctness between varieties. Chickpea is one of the principal food legumes in Ethiopia, ranking third in production next to faba bean and haricot bean. The two main types of chickpea grown in Ethiopia are *desi* (small size, coloured grain coat and angular shaped) and *kabuli* (large size, beige coloured and owl’s head shaped). [24] observed a high diversity for different grain traits among chickpea accessions. Sorghum, due to its high tolerance to drought, is the most important cereal in arid regions of Ethiopia. Its grains are small, round to oval and can be white, creamy, yellow or red. While visual discrimination can be achieved for chickpea cultivars by a well-trained expert, a majority of barley and sorghum cultivars look indistinguishable to most experts.

The main objective of the research was the identification of barley, chickpea and sorghum cultivars that have been disseminated in Ethiopia, using a miniaturized NIR spectrometer. The second objective was to compare model performances among different preprocessing techniques and choosing machine learning algorithm. Hypothesis of this study was that miniaturized NIR spectrometers can be suitable for cultivar identification. The potential applications and scalability of these tools for agricultural research, policies and industry in sub-Saharan Africa are finally discussed.

## Materials and methods

### Grain samples collection and preparation

In Ethiopia, the term improved variety is used to designate a cultivar which has been tested by breeders and evaluated for its superiority over landraces [25]. Dry cultivars of barley, chickpea and sorghum grains were collected at the Ethiopian Institute of Agricultural Research (EIAR) in June 2017. The EIAR issued the permission for scanning all cultivars used in this study. The study did not involve endangered or protected species. Grains were produced in the same year to avoid any effect of seed age. Since there is strong interest in upscaling cultivar identification using large scale household surveys, we reproduced the conditions in which grains from a seasonal harvest would be collected and dried before processing. Selected cultivars represent the most widely adopted within Ethiopia, as well as some newly developed ones. A list of cultivars included in the study is available in S1 Table. A total of 50 grains per cultivar were obtained, resulting of a sample size of N = 24 cultivars and n = 1200 barley grain samples; N = 19 cultivars and n = 950 chickpea grain samples; and N = 10 cultivars and n = 500 sorghum grain samples.

### Spectral measurements using a miniaturized NIR spectrometer

**Fig 1:**
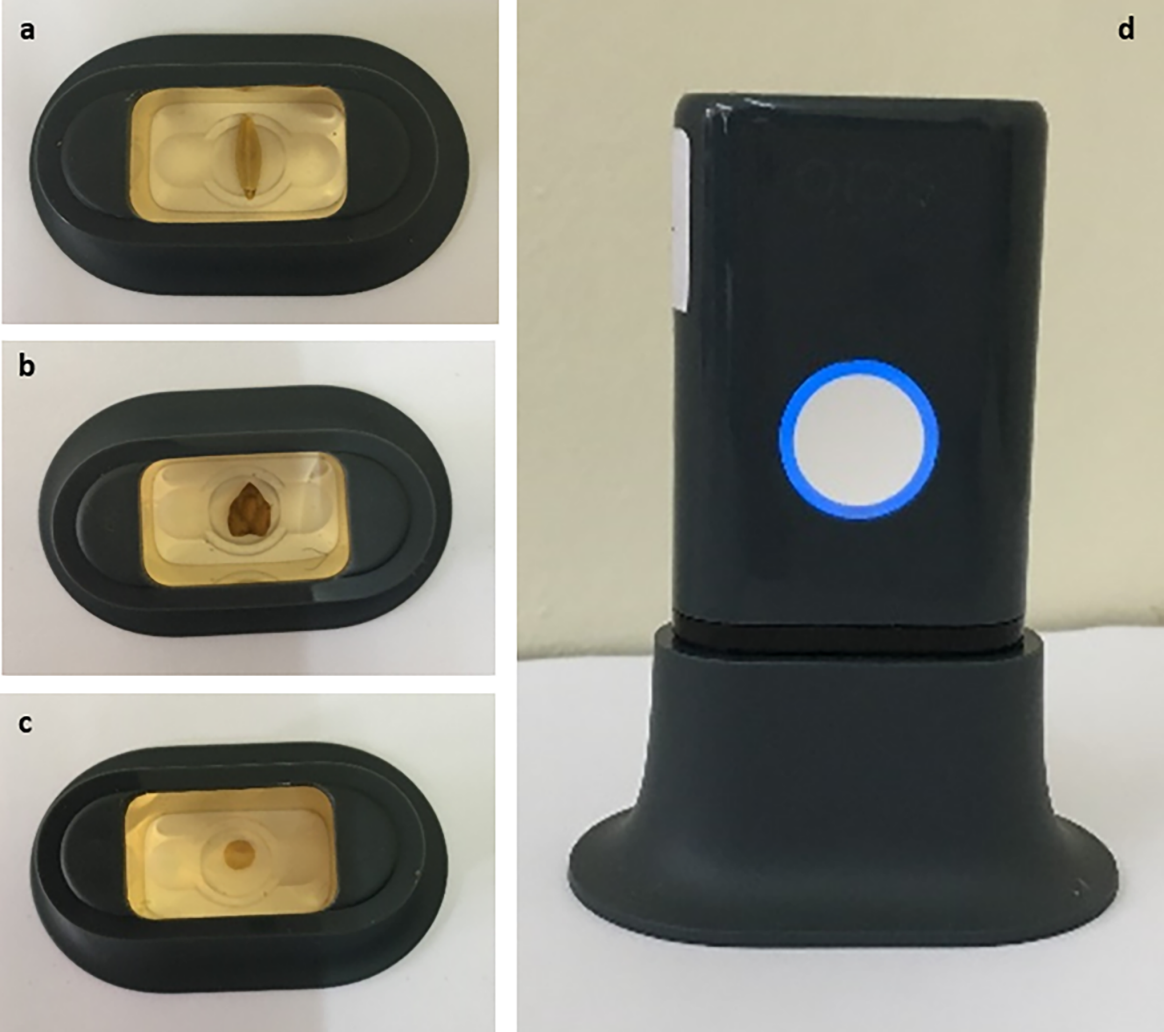
Photographs of the data collection protocol showing the position of barley, chickpea and sorghum seeds in the sample holder (a-c) as well as the SCIO in scanning mode (d).

In recent years, many companies have started to commercialize miniaturized sensors based on the emergent and promising technique of NIR spectroscopy [26]. Compared to conventional, laboratory-based spectrometers, miniaturized NIR spectrometers require minimal equipment and user involvement. For the purpose of this study, the Consumer Physics SCIO, delivered as a $1000 developer kit at the time of writing was purchased [27]. The device was operated using a smartphone application and requires an internet connection, with spectral data stored remotely. The device’s full wavelength coverage is 740–1070 nm (331 variables). All grain samples were carefully scanned in a similar position, as depicted in Fig 1.

### Pre-processing and classification algorithms

Pre-processing methods are helpful in eliminating noise generated by spectral data. Raw spectral data were thus processed using a combination of scatter corrections that include Standard Normal Variate (SNV) as well as first and second degree derivatives. The Savitzky-Golay and Gap-segment derivative smoothing filtering algorithms were also applied, using the R package prospectr [28]. In our evaluation, we then compare the performance of five algorithms to develop predictive multiclass models of grain cultivar identification: AdaBoost, Naïve Bayes, partial least squares discriminant analysis (PLS-DA), Random Forest (RF) and Support Vector Machine (SVM). These algorithms have achieved some degree of success in the domain of spectral data analysis [15, 22, 16]. However, these algorithms make different assumptions and reflect rival schools of thought [29].

### AdaBoost

Adaboost is an ensemble learning technique that uses boosting to train models sequentially [30]. A new model is trained at each round and the next model attempts to correct the errors from the previous one. Models are added until no further improvement can be made. Finally, the algorithm classifies observations by taking a weighted vote of their predictions within the different models trained.

### Naïve Bayes

Naïve Bayes uses learning as a form of probabilistic inference, in accordance with Bayes’ theorem [31]. The algorithm estimates the posterior probability P(*y* | x) of each class *y* given an object’s attributes (x). Each class is defined by its probability and by the probability distribution of each attribute among the class’ members. The algorithm makes the (naïve) assumption that the effects are independent given the cause.

### Partial least squares discriminant analysis

Partial least squares discriminant analysis (PLS-DA) is based on PLS regression and has been extensively applied in chemometrics [32]. PLS-DA extracts principal components from spectral data and ranks them given the more explained variance. Once a PLS model has been trained, the influence of predictors is captured by measuring the Variable Importance in Projection (VIP) scores derived from the PLS coefficients for the optimal set of predictors. The algorithm maximizes the covariance value between different classes and predictors are ranked by these scores and selected.

### Random Forest

Random Forest (RF) uses sets of inductive rules that are assembled to grow a forest of decision trees. First, decision trees are grown from a random subset of variables and observations. At each node (or decision rule), the attribute that on average yields the lowest class entropy across all its branches is chosen, weighted by how many observations go into each branch. Each leaf (or final node) of the tree corresponds to a single rule with a condition consisting of the conjunction of all edge labels on the decision path. A key feature of decision trees is that conditions are selected in a way that simultaneously optimizes the example distribution in all successors of a node [33]. In a RF model, each tree is constructed from a bootstrap sample of the dataset. A majority vote from the number of trees grown is used to determine the final classification of an observation.

### Support Vector Machine

The Support Vector Machine (SVM) algorithm uses the maximum margin principle to fit an optimal hyperplane between observations. Support vectors are the critical elements of the training set, as they determine the weights and thus the boundary between observations [34]. A kernel function is used as a similarity measure to construct the maximum margin models and find the optimal hyperplane. During the training process, observations are classified according to whether they lie beyond the margin, violate the margin or lie on the margin. The separating hyperplane relies on data points that lie on the margin, called the “support vectors”. SVM uses nonlinear mapping to map data into a high-dimensional feature space. In the case of non-linearly separable data, the margin assumption is relaxed, allowing some observations to violate the margin condition. Selection of a kernel function influence model performance. Here, a polynomial kernel function was used.

### Algorithm validation

**Fig 2:**
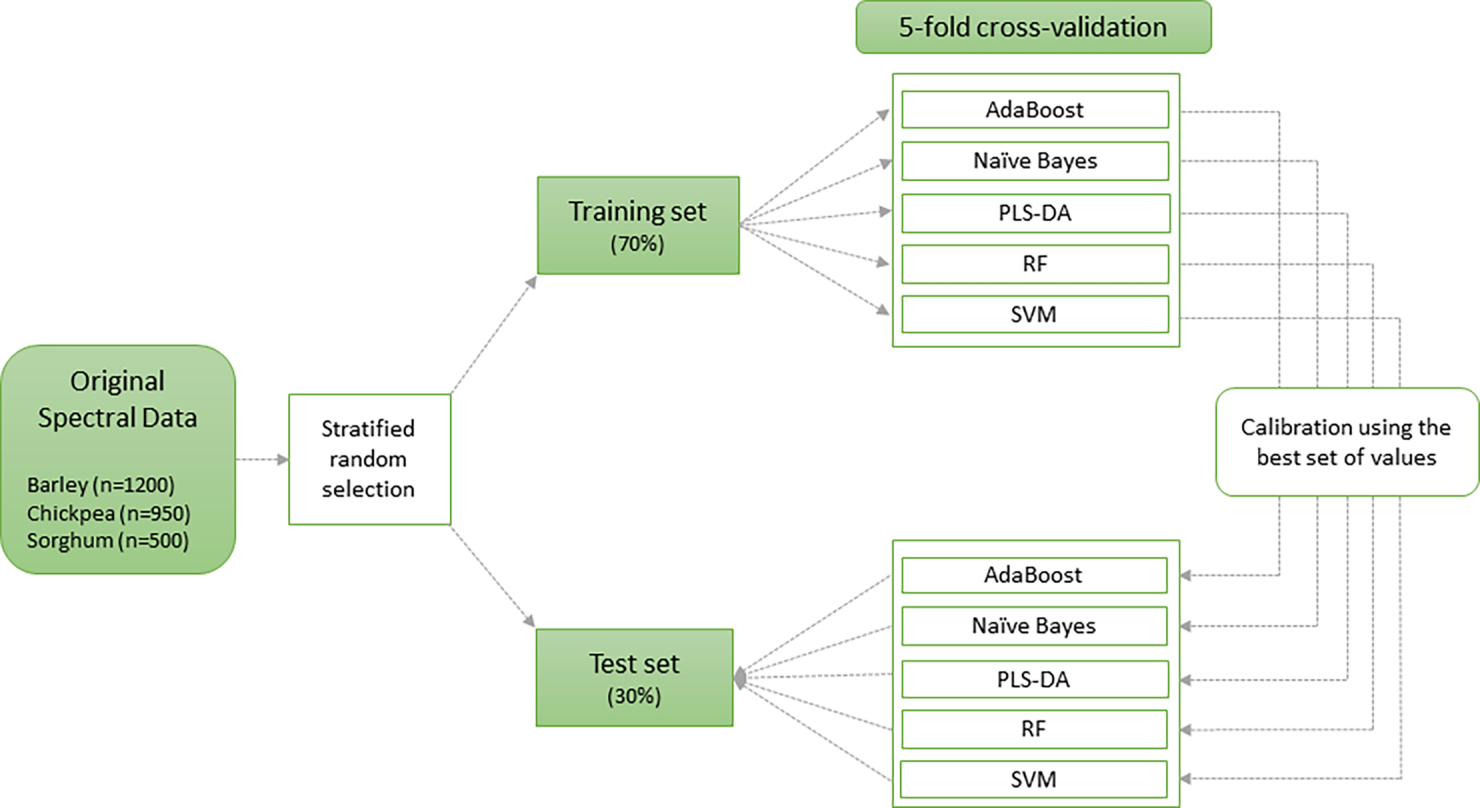
Diagram of the different calibration and validation processes used for grain cultivar identification.

In order to validate these algorithms, a two-step process was performed (Fig 2). Original spectral data were randomly split into training and test sets in a stratified way (both sets have a similar number of samples per cultivar: 35 samples in the training set, and the remaining 15 samples in the test set). Using five different algorithms, 5-fold cross-validation was executed on the training set. Cross-validation consists of partitioning the dataset into M subsets that contain the distribution of cultivars, training the model on M-1 and testing it on the remaining subset sample. Parameters were optimized during cross-validation (See S2 Table) and the best sets of values were then used to predict cultivars in the test set (hold-out sample). Overall model accuracy was assessed by the percentage of correctly classified samples in the test set. We used R [35], packages caret [36] for cross-validation on the training set as well as multiple packages for predicting the test set [37–40]. For each device, the raw spectra and 12 preprocessing techniques across five algorithms were trained and validated, totaling 390 executions (130 for each crop).

## Results

**Fig 3:**
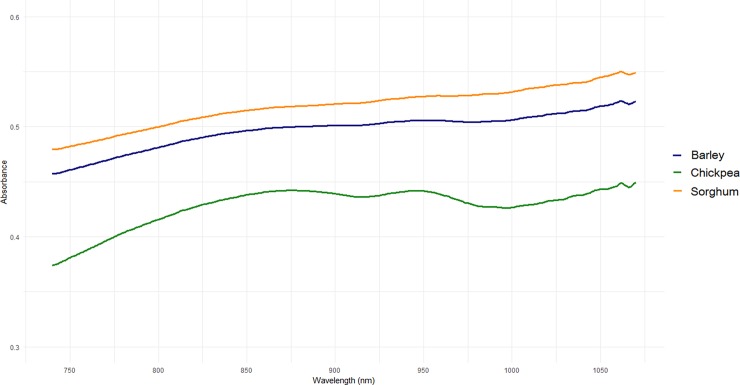
Average raw spectra of barley, chickpea and sorghum samples scanned with the SCIO (740–1070 nm).

The average raw spectra of barley, chickpea and sorghum grains obtained with the SCIO are shown in Fig 3. Chickpea grains depicted very singular spectra, while barley and sorghum grain absorbances were more closely related. Standard deviations (s.d) were comprised in the .07 to .08 range for all three crops.

### Barley cultivar identification

**Fig 4:**
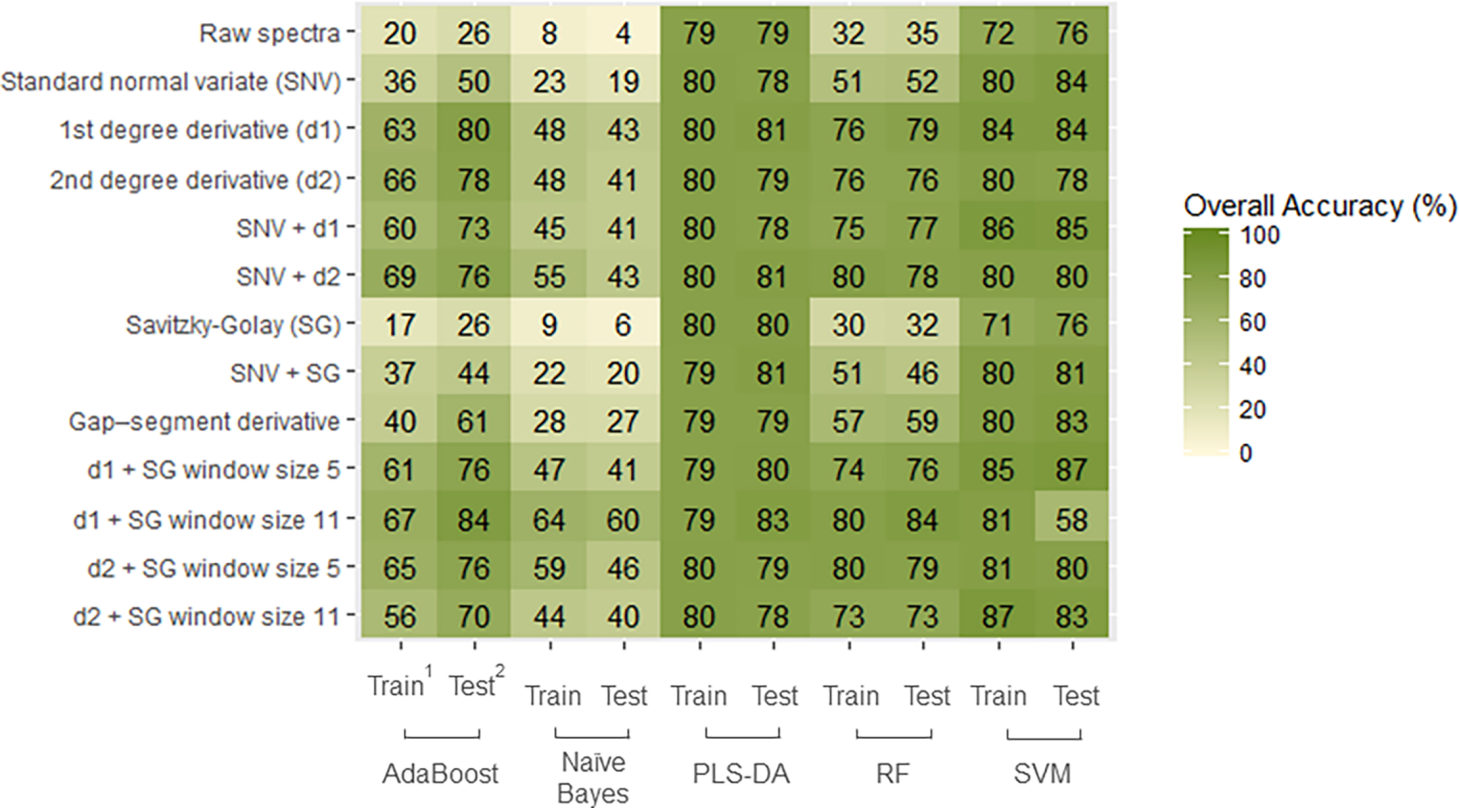
Heatmap of the overall classification accuracy of barley cultivar identification (N = 24 varieties and n = 1200 samples) using a combination of pre-processing methods (in rows) and models (in columns) on spectra collected with the SCIO. ^1^ Train represents the 5-fold cross-calibration on the training set of the samples. ^2^ Test represents the test set of the samples.

The identification results are shown in Fig 4. The SVM classifier with a first derivative and window size 5 Savitzky-Golay filtering (D1W5) yielded the highest classification accuracy (86.9% on test set) using the SCIO. Regarding the pre-processing techniques used, we observe that transformations involving the use of derivatives (d1 or d2) performed significantly better than other techniques. The SVM and PLS-DA models outperformed other algorithms.

Table 1 presents the confusion matrix from the SVM classifier with a first derivative and window size 5 Savitzky-Golay filtering (D1W5) on hold-out sample. Eight varieties achieved a perfect score, while eleven reached results above the 80% mark. The varieties Beka, HB-52 and HB-1307, believed to have reached some levels of adoption among Ethiopian farmers [4] achieved perfect accuracy while Misccal-21 was often confused with the Sabini variety. Five cultivars failed to pass the 80% mark, with important misclassification errors occurring for the Holker and Shege varieties (with Holker, a widely adopted cultivar, often misclassified here as Beka and Shege misclassified as EH-1847 and HB-1966).

**Table 1:** Confusion matrix of barley cultivars from the model that achieved the best score (SVM + d1 + SG window size 5). Overall classification accuracy is 86.9%.

	Classified as																								
	Ar	Ba	Be	Be-1	Cr	De	Di	EH-1493	EH-1847	Ex	Gr	HB-1307	HB-1533	HB-1963	HB-1964	HB-1965	HB-1966	HB-52	Ho	IB	Mi	Sa	Sh	Tr	%
**Ar**	**12**	0	0	0	0	0	0	0	0	0	3	0	0	0	0	0	0	0	0	0	0	0	0	0	80
**Ba**	0	**15**	0	0	0	0	0	0	0	0	0	0	0	0	0	0	0	0	0	0	0	0	0	0	100
**Be**	0	0	**15**	0	0	0	0	0	0	0	0	0	0	0	0	0	0	0	0	0	0	0	0	0	100
**Be-1**	0	0	0	**15**	0	0	0	0	0	0	0	0	0	0	0	0	0	0	0	0	0	0	0	0	100
**Cr**	0	0	0	0	**13**	0	0	0	0	0	0	0	0	0	0	0	0	0	0	0	0	2	0	0	87
**De**	0	0	0	0	0	**14**	0	0	0	1	0	0	0	0	0	0	0	0	0	0	0	0	0	0	93
**Di**	0	0	0	0	0	0	**15**	0	0	0	0	0	0	0	0	0	0	0	0	0	0	0	0	0	100
**EH-1493**	0	0	0	0	0	0	0	**14**	0	0	0	0	0	0	0	0	0	0	0	0	0	0	1	0	93
**EH-1847**	0	0	0	0	0	0	0	0	**14**	0	0	0	0	0	1	0	0	0	0	0	0	0	0	0	93
**Ex**	0	0	0	0	0	2	0	0	0	**13**	0	0	0	0	0	0	0	0	0	0	0	0	0	0	87
**Gr**	1	0	0	0	0	0	0	0	0	0	**14**	0	0	0	0	0	0	0	0	0	0	0	0	0	93
**HB-1307**	0	0	0	0	0	0	0	0	0	0	0	**15**	0	0	0	0	0	0	0	0	0	0	0	0	100
**HB-1533**	0	0	0	0	0	0	0	0	0	0	0	0	**11**	0	0	0	0	0	3	1	0	0	0	0	73
**HB-1963**	0	0	0	0	0	2	0	0	0	0	0	0	0	**13**	0	0	0	0	0	0	0	0	0	0	87
**HB-1964**	0	0	0	0	0	0	0	0	0	0	0	0	0	0	**15**	0	0	0	0	0	0	0	0	0	100
**HB-1965**	0	0	0	0	0	0	0	0	0	0	0	0	0	0	0	**12**	0	3	0	0	0	0	0	0	80
**HB-1966**	0	0	0	0	0	0	0	0	0	0	0	2	0	0	0	0	**10**	0	0	0	0	0	3	0	67
**HB-52**	0	0	0	0	0	0	0	0	0	0	0	0	0	0	0	0	0	**15**	0	0	0	0	0	0	100
**Ho**	0	0	7	0	0	0	0	0	0	0	0	0	1	0	0	0	0	0	**7**	0	0	0	0	0	47
**IB**	0	0	0	0	0	0	0	0	0	0	0	0	0	0	0	0	0	0	0	**15**	0	0	0	0	100
**Mi**	0	0	0	0	0	0	0	0	0	0	0	0	0	0	0	0	0	0	0	0	**11**	4	0	0	73
**Sa**	0	0	0	0	1	0	0	0	0	0	0	0	0	0	0	0	0	0	0	0	1	**13**	0	0	87
**Sh**	0	0	0	0	0	0	0	0	3	0	0	0	0	0	0	0	4	0	0	0	0	0	**8**	0	53
**Tr**	0	0	0	0	0	0	0	1	0	0	0	0	0	0	0	0	0	0	0	0	0	0	0	**14**	93

Ar: Ardu 1260 B; Ba: Bahati; Be: Beka; Be-1: Bekoji-1; Cr: Cross 41/98; De: Deribe; Di: Dimtu; EH-1493: idem; EH-1847: idem; Ex: Explorer; Gr: Grace; HB-1307: idem; HB-1533: idem; HB-1963: idem; HB-1964: idem; HB-1965: idem; HB-1966: idem; HB-52: idem; Ho: Holker; IB: IBON 174–03; Mi: Misccal-21; Sa: Sabini; Sh: Shege; Tr: Traveller.

### Chickpea cultivar identification

**Fig 5:**
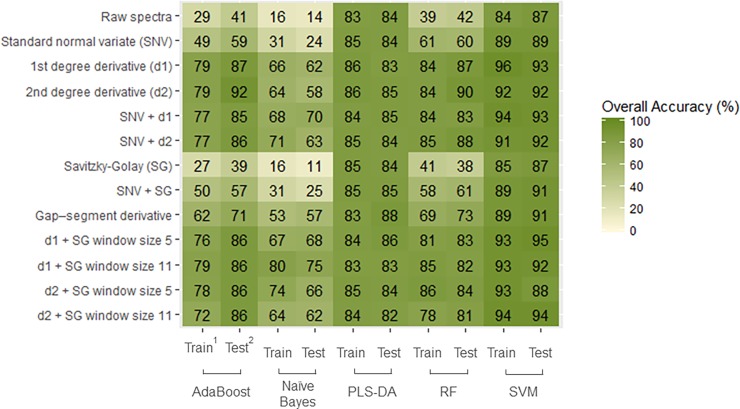
Heatmap of the overall classification accuracy of chickpea cultivar identification (N = 19 cultivars and n = 950 samples) using a combination of pre-processing methods (in raw) and models (in columns) on spectra collected with the SCIO. ^1^ Train represents the 5-fold cross-calibration on the training set of the samples. ^2^ Test represents the test set of the samples.

Chickpea cultivars display a wide range of distinct morphological attributes [24] and predictive models achieved the highest accuracy among the three crops. As demonstrated in Fig 5, SCIO data analyzed with a SVM classifier outperformed AdaBoost and PLS-DA models within all pre-processing techniques. Nearly 95% of correctly classified samples were obtained by the SVM classifier with a first derivative and window size 5 Savitzky-Golay filtering (D1W5). Table 2 shows the confusion matrix of this model. According to [4], the most adopted cultivars in Ethiopia include Arerti (6.7%), Habru (2.1%), Shasho (1.6%) and Natoli (0.3%). It is noteworthy that all cultivars are above the 80% mark, while eight cultivars were identified with perfect accuracy (including Arerti and Habru).

**Table 2:** Confusion matrix of chickpea cultivars from the model that achieved the best score (SVM + d1 + SG window size 5). Overall classification accuracy is 94.7%.

	Classified as																			
	AD	Ak	Ar	Ch	Da	Di	Du	DZ-11	DZ-4	Ej	Ha	Ho	Ma	Mi	Na	Sh	Te	Tek	Wo	%
**AD**	**14**	0	0	0	0	0	0	0	0	0	0	1	0	0	0	0	0	0	0	93
**Ak**	0	**15**	0	0	0	0	0	0	0	0	0	0	0	0	0	0	0	0	0	100
**Ar**	0	0	**15**	0	0	0	0	0	0	0	0	0	0	0	0	0	0	0	0	100
**Ch**	0	0	0	**15**	0	0	0	0	0	0	0	0	0	0	0	0	0	0	0	100
**Da**	0	0	0	0	**15**	0	0	0	0	0	0	0	0	0	0	0	0	0	0	100
**Di**	0	0	0	0	0	**14**	0	0	0	0	0	0	1	0	0	0	0	0	0	93
**Du**	0	0	0	0	1	0	**14**	0	0	0	0	0	0	0	0	0	0	0	0	93
**DZ-11**	0	0	0	0	0	0	1	**14**	0	0	0	0	0	0	0	0	0	0	0	93
**DZ-4**	0	0	0	0	0	0	0	0	**15**	0	0	0	0	0	0	0	0	0	0	100
**Ej**	0	0	0	0	0	0	0	0	0	**13**	0	0	0	0	0	2	0	0	0	87
**Ha**	0	0	0	0	0	0	0	0	0	0	**15**	0	0	0	0	0	0	0	0	100
**Ho**	0	0	0	0	0	0	0	0	0	0	0	**15**	0	0	0	0	0	0	0	100
**Ma**	0	0	0	0	0	1	0	0	0	0	0	0	**14**	0	0	0	0	0	0	93
**Mi**	0	0	0	0	0	0	0	0	0	0	0	0	0	**15**	0	0	0	0	0	100
**Na**	0	0	0	0	0	0	0	0	0	0	0	0	0	1	**13**	0	0	0	0	93
**Sh**	0	0	0	0	0	0	0	0	0	3	0	0	0	0	0	**12**	0	0	0	80
**Te**	0	0	0	0	0	0	0	0	0	1	0	0	0	0	0	0	**14**	0	0	93
**Tek**	0	0	0	0	0	0	0	0	0	0	0	0	0	0	0	0	0	**15**	0	100
**Wo**	0	0	0	0	0	0	2	0	1	0	0	0	0	0	0	0	0	0	**12**	80

AD: Acos Dubie; Ak: Akaki; Ar: Arerti; Ch: Chefe; Da: Dalota; Di: Dimtu; Du: Dubie; DZ-11: DZ-10-11; DZ-4: DZ-10-4; Ej: Ejeri; Ha: Habru; Ho: Hora; Ma: Mariye; Mi: Minjar; Na: Naatolii; Sh: Shasho; Te: Teji; Tek: Teketay; Wo: Worku.

### Sorghum cultivar identification

**Fig 6:**
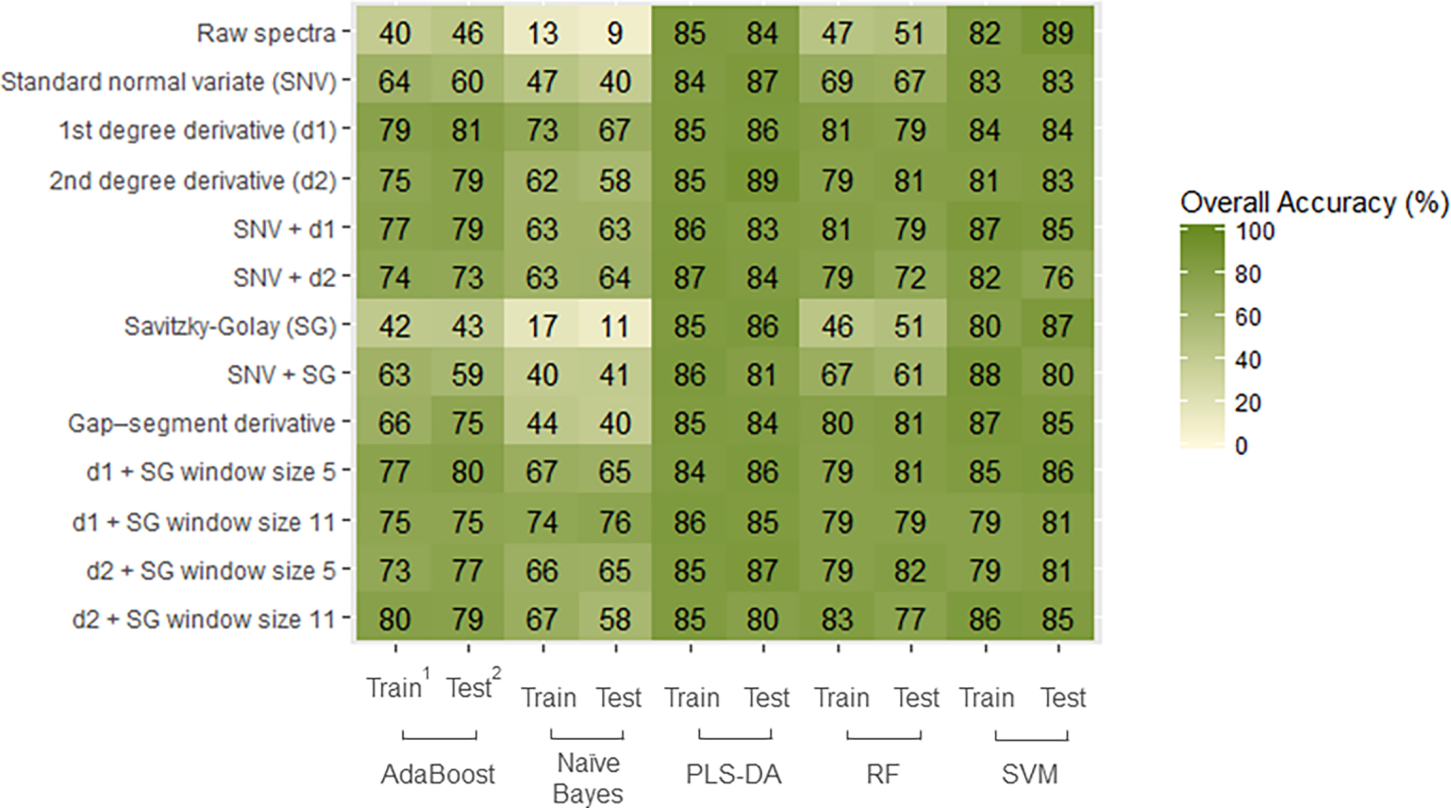
Heatmap of the overall classification accuracy of sorghum cultivar identification (N = 10 cultivars and n = 500 samples) using a combination of pre-processing methods (in raw) and models (in columns) on spectra collected with the SCIO. ^1^ Train represents the 5-fold cross-calibration on the training set of the samples. ^2^ Test represents the test set of the samples.

The different models presented in Fig 6 produced similar patterns for sorghum cultivar identification. Firstly, pre-processing techniques involving the use of derivatives (d1 or d2) proved once again effective in removing the noise present in raw data. Secondly, better performances are delivered by the SVM and PLS-DA classifiers. The overall classification accuracy was 89% for the PLS-DA model with a second degree derivative as well as for the SVM model on raw data. The confusion matrix of the latter is presented in Table 3. It can be observed that out of ten cultivars, five achieved prefect classification accuracy. The Abshir, Birhan and Teshale cultivars suffered from misclassification errors and failed to pass the 80% mark.

**Table 3:** Confusion matrix of sorghum cultivars from the model that achieved the best score (SVM + raw data). Overall classification accuracy is 88.7%.

	Classified as										
	76TI#23	Abshir	Birhan	Dekeba	Gambella 1107	Gubiye	Macia	Meko-1	Melkam	Teshale	%
**76TI#23**	**14**	0	0	0	0	0	0	0	1	0	93
**Abshir**	4	**10**	1	0	0	0	0	0	0	0	67
**Birhan**	0	4	**11**	0	0	0	0	0	0	0	73
**Dekeba**	0	0	0	**15**	0	0	0	0	0	0	100
**Gambella 1107**	0	0	0	0	**15**	0	0	0	0	0	100
**Gubiye**	0	0	0	0	0	**15**	0	0	0	0	100
**Macia**	0	0	0	0	0	0	**15**	0	0	0	100
**Meko-1**	0	0	0	0	0	0	0	**15**	0	0	100
**Melkam**	0	0	0	0	0	0	0	2	**13**	0	87
**Teshale**	0	0	0	5	0	0	0	0	0	**10**	67

## Discussion

The findings presented in this paper demonstrate the feasibility of crop cultivar identification using miniaturized NIR spectrometers. Twenty four barley cultivars, nineteen chickpea cultivars and ten sorghum cultivars were classified with an overall correct classification accuracy respectively of 89%, 96% and 87% on hold-out sample. Previous studies have also demonstrated the potential of NIR spectroscopy for cultivar identification, though discrimination was achieved under laboratory conditions and using five classes or less [13, 15]. Using a large sample size and an important number of classes to discriminate per crop, this study obtained acceptable performances (above the 80% mark) for practical applications.

Various methodologies, such as sales inquiries, expert opinion estimates and household survey questionnaires are currently employed for cultivar identification, each with their own inherent limitations [4]. In the ranking of cultivar identification survey methods, miniaturized NIR spectrometers could therefore gain in popularity. While it is impossible to determine the accuracy of other non-DNA based survey methods–household surveys, expert opinion, and sales inquiries–NIR-based cultivar identification methods offer this feature, through cross-validation. Because of this major advantage, we argue that use of miniaturized NIR spectrometers should be seen as the second-best method, following the “gold standard” of DNA fingerprinting.

It is striking that a similar pattern emerged for all three crops regarding the best pre-processing techniques and chosen algorithms. Pre-processing techniques involving the use of derivatives (d1 or d2) and a Stavitzky-Golay (SG) smooth filtering have demonstrated more effectiveness in dealing with signal noise. In addition, the PLS-DA and SVM algorithms consistently outperformed other algorithms, delivering accuracies above the 80% mark regardless of the applied pre-processing technique. By contrast, RF performed well on all crops, but only after data transformation. These results demonstrate that machine learning methods appear particularly fitted for classification problems relying on spectral data. However, contrary to other studies [15, 22, 16], we do not see evidence of the superiority of machine learning algorithms over the more traditional PLS-DA method. This could partly be explained by the narrower wavelength range of miniaturized NIR spectrometers, implying a reduction in the number of dimensions algorithms must work with.

Since the sampled grains were collected from research stations, we are unable to account for sources of environmental variability. Although phenological traits, mostly determined by crop genetics, are unlikely to be severely modified by growing conditions, it is crucial to better explore this question. This should be recognized as a limitation of the study and encourage future work to better account for potential sources of seasonal and environmental heterogeneity among cultivars. Particularly, future work should demonstrate the extent to which miniaturized NIR spectrometers can detect differences in compounds that are cultivar-specific *vs* differences in major grain components such as protein and lipid concentration. The question of how surface damage due to weathering or insects influence NIR accuracy is also an important one. The accuracy of results obtained here could certainly be improved by complementing spectral measurements with other methods, for instance computer vision [17, 41] or more advanced machine learning methods relying on ensemble models [33].

The high number of cultivars distinguished in the three crops has demonstrated the suitability of miniaturized NIR spectrometers for future survey designs. This method would allow fast, low-cost and non-destructive measurement of cultivar adoption, as well as the possibility to assess measurement errors, through cross-validation. The implications of our results are discussed in terms of four potential applications: *i*) varietal adoption surveys; *ii*) field-scale agronomic studies; *iii*) socio-economic impact assessments and *iv*) value chain quality control procedures.

First, surveys whose objective is to estimate adoption rates of cultivars at a regional or national level could lean toward a wider use of miniaturized NIR spectrometers. On the one hand, surveys relying on large scale DNA fingerprinting would benefit from complementing genetic identification with spectral measurements. Additional data collection costs could be as low as $0.1 per sample after material acquisition. Indeed, the establishment of spectral reference libraries is a critical aspect of future efforts directed toward cultivar identification and generating spectral libraries of widely adopted cultivars should be a priority. On the other hand, adoption surveys can minimize the costs of large scale DNA fingerprinting by using a coupled DNA fingerprinting / NIR spectrometry scheme on a stratified sub-sample. Crop genetic identity can then be used to calibrate spectra obtained from NIR spectrometers on the rest of the sample. The costs of cultivar identification would thus largely decrease. The minimum number of grains and cultivars to be included in a reference library are crop- (variability in grain characteristics) as well as scale-specific. Libraries that include too few samples increase the chance of not being representative of the prediction sample.

**Fig 7:**
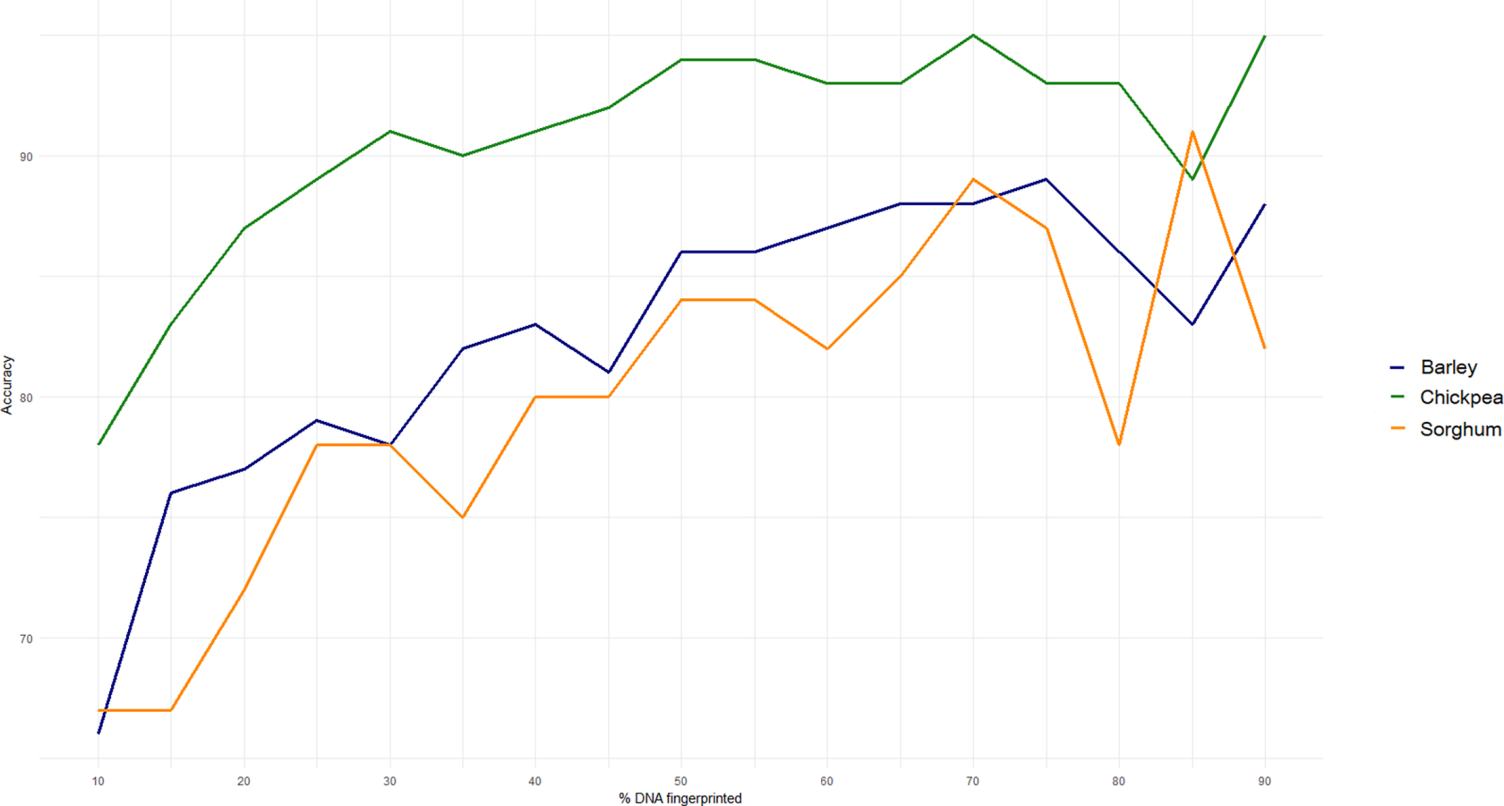
Accuracy of multiclass predictive models of barley, chickpea and sorghum cultivar identification on test set given the percentage of DNA fingerprinted grains used as calibration library.

To this effect, Fig 7 provides interesting insights. Based on the data presented in this study, the plot compares the accuracy of prediction per crop given the percentage of the sample DNA fingerprinted. Assuming that a random stratified sample is DNA fingerprinted, and used as a calibration library, it is observed that cost-effectiveness would be reached by DNA fingerprinting 65% of barley grains, half of the chickpea grains and 70% of sorghum grains. Beyond these levels, the additional costs of DNA analysis are probably not worth the gains in accuracy.

Second, studies which seek to assess the on-farm impact of specific varieties could greatly benefit from the use of miniaturized NIR spectrometers. Field-scale studies usually complement agronomic trials, realized under controlled conditions [42, 3, 43]. Although some caution is observed in the literature regarding the extrapolation from plot-level experiments to farm fields [44], field-scale agronomic studies remain scarce [45]. Miniaturized NIR spectrometers could be a crucial component of field-scale research. Evidence could be further generated on the suitability of a cultivar to different environments as well as the contributing factors to observed yield gaps.

Third, additional pieces of evidence often need to be gathered on the socio-economic aspects of varietal adoption. While several research designs are possible, these studies typically rely on two or more waves of data collection [46–48]. In field areas with high varietal diversity, one can imagine a research design where all grains would be DNA fingerprinted and NIR scanned during a baseline survey, while the follow-up survey could generate spectral data only and use the calibrations obtained at baseline survey to estimate cultivar adoption. This would greatly reduce survey costs while also providing statistical information on the accuracy of cultivar identification. For both field-scale agronomic studies and socio-economic studies, it is usually not necessary to identify all cultivars, but only the cultivars of interest. Thus, our finding that a total of eight barley cultivars, nine chickpea cultivars and five sorghum cultivars achieved perfect classification accuracy contributes in establishing the validity of the method for binary classification models (cultivar of interest *vs* other).

Fourth, these results are useful for governments and institutions interested in establishing grain certification procedures and grain quality controls [49, 19]. Such a policy framework would undoubtedly enhance the quality of commercially-available grains while providing positive feedback for smallholder farmers. Certainly, miniaturized NIR spectrometers are effective instruments to perform quality controls along the value chain. The considerable amount of data available on grain production units could facilitate the calibration of accurate varietal identification models.

## Conclusion

Crop cultivar identification is a major preoccupation for both research and policy and is necessary information that needs to be obtained at various stages of the value chain. The time-consuming and costly nature of DNA fingerprinting can restrict the ability of practitioners to obtain valid measurements. Our results provide empirical evidence of the applicability of miniaturized NIR spectrometers and machine learning methods for crop cultivar identification. Interactions between preprocessing techniques and learning methods that contribute to model performance were explored and a reproducible R code can be used to replicate the analysis presented in this article. These results certainly require further validation and future research should seek to better understand site-specific variations, grain surface damages as well as grain moisture content status. Finally, as an added feature of this study, we provide an R code that allows grain varietal identification by testing the full combination of pre-processing methods and algorithms (S2 File). Cross-validation on training set and prediction on test set are outputted as a CSV file. Certainly, miniaturized NIR spectrometers and machine learning tools have the ability to shed a different light on cultivar identification methods.

## Supporting information

S1 Table Cultivars of barley, chickpea and sorghum tested using miniaturized NIR spectrometers. (DOCX)

S2 Table Description of tuned parameters, R package used and average execution time of all models on both devices. (DOCX)

S1 File Raw datasets. Materials submitted for reproducibility.
(XLSX)

S2 File R code. Material submitted for reproducibility. The code allows grain cultivar identification by testing the full combination of pre-processing methods and algorithms presented here.
(R) Click here for additional data file.
