# Supplementary material for: Evaluation of a miniaturized NIR spectrometer for cultivar identification: The case of barley, chickpea and sorghum in Ethiopia
Source: PLoS One. 2018 Mar 21;13(3):e0193620. doi: 10.1371/journal.pone.0193620 (PMC5862431; doi:10.1371/journal.pone.0193620)
Supplement: S1 Table — (DOCX) [file pone.0193620.s001.docx]

Table S.1. Cultivars of barley, chickpea and sorghum released by the national agricultural research system of Ethiopia and tested using miniaturized NIR spectrometers.

| Crop | Name | Type | Year of release |
| --- | --- | --- | --- |
| Barley | Ardu 1260 B | Food | 1986 |
|  | Bahati | Malt | 2011 |
|  | Beka | Malt | 1976 |
|  | Bekoji-1 | Malt | 2010 |
|  | Cross 41/98 | Food | 2012 |
|  | Deribe | Food | 2010 |
|  | Dimtu | Food | 2001 |
|  | EH-1493 | Food | 2012 |
|  | EH-1847 | Malt | 2011 |
|  | Explorer | Malt | 2017 |
|  | Grace | Malt | 2013 |
|  | HB-1307 | Food | 2006 |
|  | HB-1533 | Malt | 2004 |
|  | HB-1963 | Malt | 2016 |
|  | HB-1964 | Malt | 2016 |
|  | HB-1965 | Malt | 2017 |
|  | HB-1966 | Malt | 2017 |
|  | HB-52 | Malt | 2001 |
|  | Holker | Malt | 1979 |
|  | IBON 174-03 | Malt | 2012 |
|  | Misccal-21 | Malt | 2006 |
|  | Sabini | Malt | 2011 |
|  | Shege | Food | 1995 |
|  | Traveller | Malt | 2013 |
| Chickpea | Acos Dubie | Kabuli | 2009 |
|  | Akaki | Desi | 1995 |
|  | Arerti | Kabuli | 2000 |
|  | Chefe | Kabuli | 2004 |
|  | DZ-10-4 | Desi | 1974 |
|  | DZ-10-11 | Desi | 1974 |
|  | Dalota | Desi | 2013 |
|  | Dimtu | Desi | 2016 |
|  | Ejeri | Kabuli | 2005 |
|  | Habru | Kabuli | 2004 |
|  | Hora | Kabuli | 2016 |
|  | Mariye | Desi | 1985 |
|  | Minjar | Desi | 2010 |
|  | Naatolii | Desi | 2007 |
|  | Shasho | Kabuli | 2000 |
|  | Teji | Kabuli | 2005 |
|  | Teketay | Desi | 2013 |
|  | Worku | Desi | 1994 |
| Sorghum | 76TI#23 | / | 1979 |
|  | Abshir | / | 2000 |
|  | Birhan | / | 2002 |
|  | Dekeba | / | 2012 |
|  | Gambella | / | 2007 |
|  | Gubiye | / | 2000 |
|  | Macia | / | 2007 |
|  | Meko | / | 1997 |
|  | Melkam | / | 2009 |
|  | Teshale | / | 2002 |
