# Supplementary material for: Evaluation of a miniaturized NIR spectrometer for cultivar identification: The case of barley, chickpea and sorghum in Ethiopia
Source: PLoS One. 2018 Mar 21;13(3):e0193620. doi: 10.1371/journal.pone.0193620 (PMC5862431; doi:10.1371/journal.pone.0193620)
Supplement: S2 Table — (DOCX) [file pone.0193620.s002.docx]

Table S.2. Description of tuned parameters, R package used and average execution time of all models on both devices

| Learning method | Parameters tuned during cross-validation on train set^1^ | R package used for prediction on test set | Average execution time^2^ |
| --- | --- | --- | --- |
| AdaBoost | Number of iterations per boosting (mfinal); weight updating coefficient (coeflearn) | adabag | 28 hours |
| Naïve Bayes | Laplace correction (fL); kernel density estimate (usekernel) | klaR | 3 hours 28 minutes |
| PLS-DA | Number of components (ncomp) | caret | 50 minutes |
| Random Forest | Number of predictor sampled at each split (mtry); number of trees (ntree) | randomForest | 9 hours 20 minutes |
| SVM | C; degree; gamma | e1071 | 15 hours |

^1^ Using the train () function from the R caret package; ^2^ For both devices, using the following hardware specifications: Intel Core i5-6200U CPU, 2.40 GHz processor and 8 GB of RAM.
